# On the Treatment of Tic Douloureux

**Published:** 1818-12

**Authors:** P. Leslie

**Affiliations:** Member of the Royal College of Surgeons, London; late of the Bombay Medical Establishment.


*For the London Medical and Physical Journal.*

### *On the Treatment of Tic Douloureux;* by P. Leslie, M.D.

Member of the Royal College of Surgeons, London; late of the Bombay Medical Establishment.

UPON looking over some of your late Journals, I observe, in the Report of Diseases for February last, the extract of belladona had been used, with a good deal of success, by Mr. Bailie in tic douloureux. I confess it is a medicine I have never used in that complaint; therefore cannot speak of its efficacy from my own observation, although, from ten years' residence in India and Persia, I have had many opportunities of seeing tic douloureux in its worst state, as it is particularly prevalent among the natives of these countries. In such cases, I have always prescribed small doses of the submuriate of mercury and opium, two grains of the former and half a grain of the latter, three times a-day, in the form of a pill; and, I think I may venture to affirm, generally with success. I have seldom found the painful affection to continue after the mouth became slightly affected; but, in most cases, relief has been obtained before ten grains of the submuriate, and five of opium, had been taken.

Since my residence in this country, I have made use of the above practice, and have found it equally advantageous in four cases to which I have applied it; and more particularly in the last of these, which was of a gentleman residing in this neighbourhood, who had suffered severely for two or three years. At times he was almost driven to a state of distraction from it (to use his own expression), and had used various remedies without receiving any permanent relief; and before he had taken twelve grains of the submuriate, and six grains of opium, the pain subsided; nor has he had any return of the complaint since, which is upwards of six months ago.

*Pontesford, near Shrewsbury;*

*Nov. 4th,* 1818.

